# Accuracy and Reliability of Internet Resources for Information on Monoclonal Gammopathy of Undetermined Significance—What Information Is out There for Our Patients?

**DOI:** 10.3390/cancers13184508

**Published:** 2021-09-07

**Authors:** Emma Pauline Kreutzer, Sandra Sauer, Mark Kriegsmann, Henrike Staemmler, Gerlinde Egerer, Katharina Kriegsmann

**Affiliations:** 1 Department of Hematology, Oncology and Rheumatology, University Hospital Heidelberg, 69120 Heidelberg, Germany; EmmaPaulineElisabeth.Kreutzer@med.uni-heidelberg.de (E.P.K.); Sandra.Sauer@med.uni-heidelberg.de (S.S.); henrike.staemmler@web.de (H.S.); Gerlinde.Egerer@med.uni-heidelberg.de (G.E.); 2 Institute of Pathology, University Hospital Heidelberg, 69120 Heidelberg, Germany; Mark.Kriegsmann@med.uni-heidelberg.de

**Keywords:** monoclonal gammopathy of undetermined significance (MGUS), online health information, patient education

## Abstract

**Simple Summary:**

In the current analysis, we provide a comprehensive evaluation of monoclonal gammopathy of undetermined significance (MGUS)-related health information available online. An analysis of general and patient- (user-) focused quality, readability, and content of websites and videos was performed. It revealed a generally medium to low quality of internet resources. Therefore, understandability, informative value, and support in a decision-making process can be attributed to single websites/videos only. Our study clearly highlights the risk of misinformation by insufficient, incorrect, misleading, and out-of-date information. Knowing what content is assessable to patients online may help clinicians to educate their patients and actively address misinformation.

**Abstract:**

Background: Online information gathering can increase patients’ engagement in decision-making. The quality of online resources available for monoclonal gammopathy of undetermined significance (MGUS) was evaluated. Methods: 900 websites from Google, Bing, Yahoo, and 150 YouTube videos were assessed. Results: The websites did not differ regarding their search rank or between the search engines. The median time since last update was 24 months. The 86 unique websites showed a medium to poor general quality (JAMA score 3/4, only 8.1% websites with a valid HON certificate). The patient- (user-) focused quality was poor (sum DISCERN score 27/80 points). The reading level was difficult (11th US school grade). The content level was very low (13/50 points). 12.8% of websites contained misleading/wrong facts. Websites provided by scientific/governmental organizations had a higher content level. For the 61 unique videos, the median time since upload was 34 months. The videos showed a medium general quality (HON Foundation score). The patient- (user-) focused quality was poor (sum DISCERN score 24 points). The content level was very low (6 points). Conclusion: MGUS-relevant online sources showed a low quality that was provided on a high reading level. Incorporation of quality indices and regular review of online content is warranted.

## 1. Introduction

Monoclonal gammopathy of undetermined significance (MGUS) is a clonal, premalignant plasma cell or B-cell disorder, characterized by the presence of monoclonal protein and absence of multiple myeloma or lymphocytic/lymphoplasmacytic malignancies [1,2]. Given the high prevalence, around 3% in persons ≥50 years and around 5% in persons ≥70 years, MGUS is of particular clinical interest [3]. Depending on the subtype of MGUS, there is a considerable risk of progression to multiple myeloma (MM), lymphoplasmacytic malignancy, or other serious disorders, such as light chain amyloidosis of 0.3% to 1% per year [2]. Upon an extensive initial evaluation, indications for follow-up examinations on a regular basis are given in all patients to detect progression. For patients, this implicates a constant fear of facing cancer, considerable anxiety, and concerns.

During the course of the disease, patients often seek MGUS-related information online and address it during the consultation with a physician. Therefore, knowing what content is assessable to patients online may help clinicians to educate their patients, actively address misinformation, and reduce their concerns and fears [4].

The variety of online sources on health- and disease-related topics published on or delivered by the World Wide Web is enormous: websites provided by academic or non-academic institutions, social media, blogs and support forums, patient portals, news aggregators, etc. [5]. The 2019 survey by Eurostat, the statistical office of the European Union, revealed that “one in two citizens (53%) aged 16–74 reported that they sought online health information related to injury, disease, nutrition, improving health or similar” during the last three months [6]. As shown by previous analyses, online information gathering can increase patients’ competence with and engagement in health decision-making strategies and health maintenance [7,8,9]. Despite these advantages, there are several concerns regarding an overabundance of irrelevant, incomplete, or invalid information, anxiety and poor compliance resulting from a false interpretation of written information, destruction by unrelated information, and selective patients’ perception that satisfies the search intention [10,11,12,13,14]. One might argue that high-quality diagnostic and clinical guidelines and academic articles are also available online. However, these sources of information are not always freely available and are written at a high reading level, which negatively influences the accessibility and patients’ understanding.

The aim of the current analysis was to evaluate the quality of online resources available for MGUS. This was achieved by a score-based assessment of general and patient-focused quality, readability, and content of websites and YouTube videos.

## 2. Materials and Methods

### 2.1. Website and Video Search and Selection Strategy

Written online health information on MGUS in English was analyzed. North American access for the search engines Google, Bing, and Yahoo was used. Searches were performed on 18–26 March 2020 after the removal of the web browser cookies and history. The three search terms “monoclonal gammopathy”, “monoclonal gammopathy of undetermined significance” and “MGUS” were applied. For each search term, the first 100 search hits in each search engine were selected for further evaluation, resulting in 300 websites per search engine and 900 overall initial websites.

To identify videos with health information on MGUS, the online video-sharing platform YouTube was searched for the three terms. Searches were performed on 28–31 March 2020. For each search term, the first 50 search hits were selected for further evaluation, resulting in 150 overall initial videos.

Websites and videos with no relevance to MGUS (e.g., only including the search term in a bullet list), requiring fees or registration, using other languages than English or scientific journal articles intended for professionals were defined as not eligible.

For website evaluation, in a first step, websites not meeting the eligibility criteria and duplicates were excluded for each search engine separately and an evaluation by the search engine was performed. In a second step, the final overall list of websites was created by removing the duplicates between the search engines. These websites (called “unique”) are meant to represent the primarily assessable body of information to the patients.

### 2.2. Assessed Variables and Scores

For detailed evaluation, general website and video information, general quality of medical information online, patient- (user-) focused quality of medical information online, readability, and entity-related content were assessed (Table S1) [15].

General information on websites included the search rank of the respective search engine, paid advertising website, URL (Uniform Resource Locator), host continent, website category (scientific/governmental, e.g., the National Cancer Institute, Bethesda, United States; foundation/advocacy, e.g., the Leukemia Foundation, Brisbane, Australia; news/media, industry/for-profit; personal commentary/blog), update and access date.

General information on videos included the search rank, URL/title, host continent, video category (identical to website category), upload and access date, video duration, number of views, likes, dislikes, and comments. The viewing rate was calculated as: views/days since upload. The engagement rate was calculated as: (likes + dislikes + comments)/views.

The general quality of medical information online was evaluated by the Health on the Net (HON) Foundation certificate/score and the Journal of the American Medical Association (JAMA) score. HON is an international not-for-profit, non-governmental organization that promotes transparent and reliable health information online. Providers of health information websites can certify their website by the HON Foundation [16,17]. As the HON certificate applies for websites only, a step-by-step evaluation of videos was performed according to the eight HON Foundation principle criteria (authority, complementarity, confidentiality, attribution, justifiability, transparency of authorship, transparency of sponsorship, honesty in advertising, and editorial policy; minimum points 0, maximum points 8). The achieved points translate into a low (0–2 points), medium (3–5 points), and high (6–8 points) quality category according to the HON Foundation. The JAMA score evaluates a series of four criteria (authorship, attribution, disclosure, and currency; minimum points 0, maximum points 4) and aims to assess, control, and assure the quality of medical information on the internet [18].

The patient- (user-) focused quality of medical information online was evaluated by the DISCERN score, an instrument for judging the quality of written consumer health information on treatment choices. It addresses the following questions: is the publication reliable? (section 1, item 1–8); how good is the quality of information on treatment choices? (section 2, item 9–15); overall rating of the publication (item 16) [19]. A minimum of 1 point (not addressed) and a maximum of 5 (fully addressed) points can be achieved per DISCERN score item, resulting in a minimum of 16 (1 point × 16 items) and a maximum of 80 (5 points × 16 items) achievable points per website/video. As MGUS requires follow-up rather than treatment, section 2 was modified as follows: item 9—how treatment works—modified to the significance of MGUS diagnosis, item 10—benefits of treatment—modified to benefits of follow-up, item 11—risks of treatment—modified to disadvantages of follow-up, item 12—no treatment options—modified to risks of no follow-up, item 13—quality of life—modified to uncertainty associated with follow-ups, item 14—other treatment options—modified to variation of follow-up intervals, item 15—shared decision making—not modified. To ensure the best possible objectivity, the evaluation of websites and videos according to the DISCERN was performed by two observers.

The readability of websites was assessed according to the Flesch Reading Ease score and the Flesch Kincaid Grade level. The Flesch Reading Ease score was calculated as: 206.835 − (1.015 × average sentence length) − (84.6 × average number of syllables per word) and ranges between 0–30 (very difficult to understand text) and 90–100 (very easy to understand text) [20]. The Flesch Kincaid Grade level uses a modified Flesch Reading Ease formula ((0.39 × average sentence length) + (11.8 × average number of syllables per word) − 15.59) to produce a grade-level score according to the US school grade that is needed to understand a text [21].

The entity-related content was assessed according to 50 key facts on MGUS derived from current guidelines and addressing the categories definition, symptoms, risk factors, evaluation, management, outcome, and risk of progression (Table S2) [1,2,22]. 0 (not addressed), 0.5 (partially addressed), and 1 (fully addressed) points were achievable per key fact items, resulting in a minimum of 0 (0 points × 50 items) and a maximum of 50 (1 point × 50 items) achievable overall content points per website/video. If applicable, misleading, and wrong facts were recorded and classified according to the key fact category.

### 2.3. Statistical Analysis

Statistical analysis was performed in R studio (R version 4.0.2, 2020-06-22, The R Foundation for Statistical Computing). Data were presented as absolute numbers and percentages, medians and ranges as well as the means and standard deviations (SD) as appropriate.

To analyze contingency tables Fisher’s exact was used. The distribution of ordinal scaled variables was compared by the Mann–Whitney U test (two groups) or the Kruskal–Wallis H test (more than two groups). To identify differences between group means for continuous variables, comparisons were performed with unpaired two-tailed Student’s *t*-tests (two groups) or analysis of variance (ANOVA, more than two groups). Linear regression was performed to investigate the correlation between the website search rank and the sum DISCERN score, sum key facts score, and time since update.

Inverse Kaplan–Meier curves were chosen to demonstrate the proportion of website/video updates by time. An event represents a website update. The time to event was calculated as a difference in months between the update/upload and access date. Websites not indicating an update date were excluded from the Kaplan–Meier curve representation.

Unsupervised hierarchical clustering (R package ‘ComplexHeatmap’) by the website and DISCERN or key fact score items was performed in order to identify an association between website category and addressed items.

A *p* ≤ 0.05 was considered statistically significant.

## 3. Results

### 3.1. Characterization of Websites on MGUS

#### 3.1.1. Search and Selection Results

The initial search with three search terms resulted in 300 hits per search engine and, therefore, overall 900 websites. The removal of duplicates between the search terms and websites not meeting the eligibility criteria resulted in 53, 66, and 59 websites for Google, Bing, and Yahoo, respectively. The removal of duplicates between the three search engines resulted in 86 unique MGUS-relevant websites (Table 1).

#### 3.1.2. Characteristics According to Search Engine

In order to identify differences between the three search engines a characterization and comparison of websites found on Google (*n* = 53), Bing (*n* = 66), and Yahoo (*n* = 59) was performed in the first step (Table S3).

We could not identify any statistically significant differences regarding the general website information. There was one promoted website on each search engine in terms of paid advertising. A comparison of websites with an indicated update date regarding the time since last update did not show any statistically significant differences (median time since last website update: Google 16 months, Bing 21 months, Yahoo 21 months, *p* = 0.720, Figure S1D).

The MGUS-relevant websites identified on Google, Bing and Yahoo also did not significantly differ in terms of the general quality of medical information online (availability of the HON Foundation certificate, JAMA score), patient- (user-) focused quality of medical information online (DISCERN score), readability (Flesch Reading Ease score, Flesch Kincaid Grade Level), and MGUS-related content (key fact score, misleading/wrong facts).

Moreover, no correlation between the search rank and the sum of the DISCERN score, the sum of the key fact score, or the time since upload was identified for any search engine (Figure S1A–C).

#### 3.1.3. Quality and Content of Unique Websites

**Figure 1:**
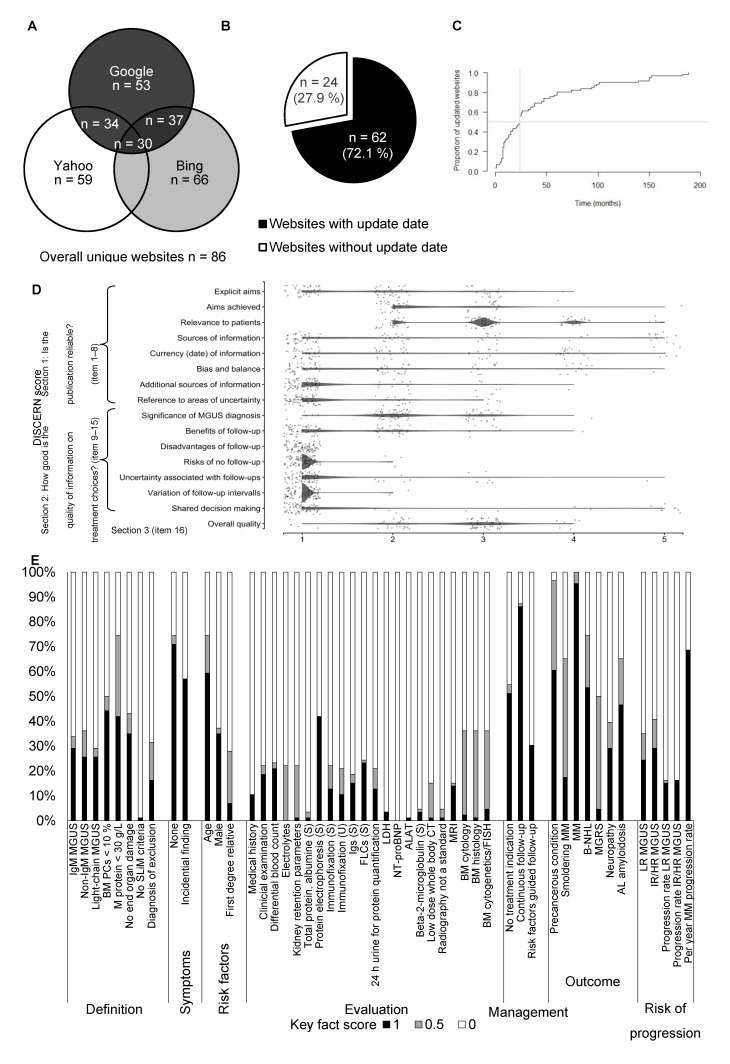
Characterization of unique websites. (**A**) Venn diagram indicating the number of duplicate overlaps between the search engines. Overall, 86 unique websites were identified. (**B**) Pie chart showing the proportion of websites with and without indicated update date. (**C**) Inverse Kaplan–Meier curve shows the proportion of website updates by time. Only websites with an indicated update date were included (*n* = 62). (**D**) Scatter dot plot shows the score result reached by every single website (*n* = 84) for each item of the DISCERN score. The categorial item scoring ranges between 1 (not addressed/fulfilled) and 5 (fully addressed/fulfilled). To avoid a visual overlap, the dots were spread around the respective score category. (**E**) For each of the 50 key fact items, the proportion of websites (*n* = 86) fully (1), partially (0.5), or not (0) addressing the respective contents is shown. The results are grouped by key fact category.

Eighty-six unique MGUS relevant websites, representing the primarily assessable body of information to the patients, were identified (Figure 1A) and subjected to an elaborate characterization (Table 2).

The most websites were provided either by a scientific/governmental organization (*n* = 38, 44.2%) or a foundation/advocacy (*n* = 31, 36.1%). Around 20% of websites were provided by news/media, industry/for-profit organizations, or by single individuals in terms of a personal commentary/blog. The majority of websites originated from North America (*n* = 68, 79.1%), followed by Europe (*n* = 11, 12.8%). Sixty-two websites (72.1%) indicated an upload/update date (Figure 1B). The median time since upload/last update of those websites was 24 months (Figure 1C).

The general quality of medical information online was acceptable in terms of the JAMA score: of 4 maximum achievable points on authorship, attribution, disclosure, and currency, in median 3 (range 0–4) points were addressed by the evaluated websites. However, only a few websites had a valid HON Foundation certificate (*n* = 7, 8.1%).

The patient- (user-) focused quality of medical information online assessed by the DISCERN score was rather poor: of 16 minimum and 80 maximum possible points, in median 27 (range 16–43) points were achieved by the evaluated websites. While the items on the reliability of the provided information were at least addressed (section 1), information on treatment choices/follow-up of MGUS (section 2) was provided by very few websites (Figure 1D, Figure S2B, Table S4).

The readability according to the mean Flesch Reading Ease score of the evaluated websites was difficult (mean 48, SD 10). The mean Flesch Kincaid Grade level was 11 (SD 3), indicating that at least an 11th US school grade is necessary to understand the website content.

The MGUS related content was evaluated by a set of 50 very detailed key facts (Figure 1E, Figure S2C). Of 50 maximum possible points, in median 13 (range 3–37) points were achieved. Approximately one-third of the evaluated websites at least partially addressed the aspects of MGUS diagnosis criteria. However, the absence of recently established SLiM criteria (clonal bone marrow plasma cells <60%, involved/uninvolved serum free light chain ratio <100, ≤1 focal lesions on magnetic resonance imaging) at MGUS first diagnosis were mentioned only by one website. A detailed enumeration of analyses to be performed for MGUS evaluation was not part of the content provided by most websites. The absence of symptoms, possible risk factors, management in terms of continuous follow-up, possible progression including progression rates into MM, B-cell non-Hodgkin lymphoma, and other associated conditions were frequently part of the content provided by the evaluated websites. Eleven websites (12.8%) containing, overall, 40 misleading/wrong facts were identified (Table 3).

Ten misleading/wrong facts (25.0%) related to the definition of MGUS diagnosis. Exemplarily, MGUS was falsely described to be associated with symptoms of end-organ damage—which in fact contradicts the diagnosis criteria of MGUS. Moreover, misleading information on risk factors of MGUS (*n* = 9, 22.5%), as a strong association with infections or environmental toxins, was provided. The most misleading/wrong facts related to the management of MGUS (*n* = 15, 37.5%). In this regard, misleading information on a treatment indication given an MGUS diagnosis was provided. Moreover, partially absurd suggestions on nutrition and diet were identified.

A data set containing the original data on selected quality, content scores, and citations of misleading/wrong facts obtained from the unique websites is provided along with this manuscript (Table S5).

#### 3.1.4. Website Characteristics According to Category

In order to identify differences between the website sources, a characterization and comparison of websites by providers was performed (scientific/governmental *n* = 38, foundation/advocacy *n* = 31, news/media *n* = 13, Table 4). Due to the low case number of websites, the industry/for-profit (*n* = 2) and personal commentary/blog (*n* = 2) categories were not included.

Although the median time since last update was shorter in scientific/governmental website providers (13 months) compared to foundations/advocacies (25 months) or news/media (24 months), this difference was not statistically significant (*p* = 0.600, Figure S2A).

The MGUS-relevant websites of different providers also did not significantly differ in terms of the general quality of medical information online (availability of the HON Foundation certificate, JAMA score), patient- (user-) focused quality of medical information online (DISCERN score, Figure S2B), and readability (Flesch Reading Ease score, Flesch Kincaid Grade Level).

However, websites provided by scientific/governmental institutions have a significantly higher median sum key fact score (17, range 4–37) compared to foundations/advocacies (11, range 5–35) or news/media (11, range 5–33), (*p* = 0.022). This is also reflected by the hierarchically clustered heatmap, that shows clusters of websites provided by scientific/governmental institutions along with the most fully and partially addressed key fact items (Figure S2C).

### 3.2. Characterization of Videos on MGUS

#### 3.2.1. Search and Selection Results

The initial YouTube search with 50 hits per search term resulted in overall 150 videos. The removal of duplicates between the search terms and videos not meeting the eligibility criteria resulted in 61 MGUS relevant videos (Table 1).

#### 3.2.2. Quality and Content of Unique Videos

The 61 unique MGUS relevant videos were assumed as the primarily assessable body of information and subjected to an elaborate characterization (Table 2).

**Figure 2:**
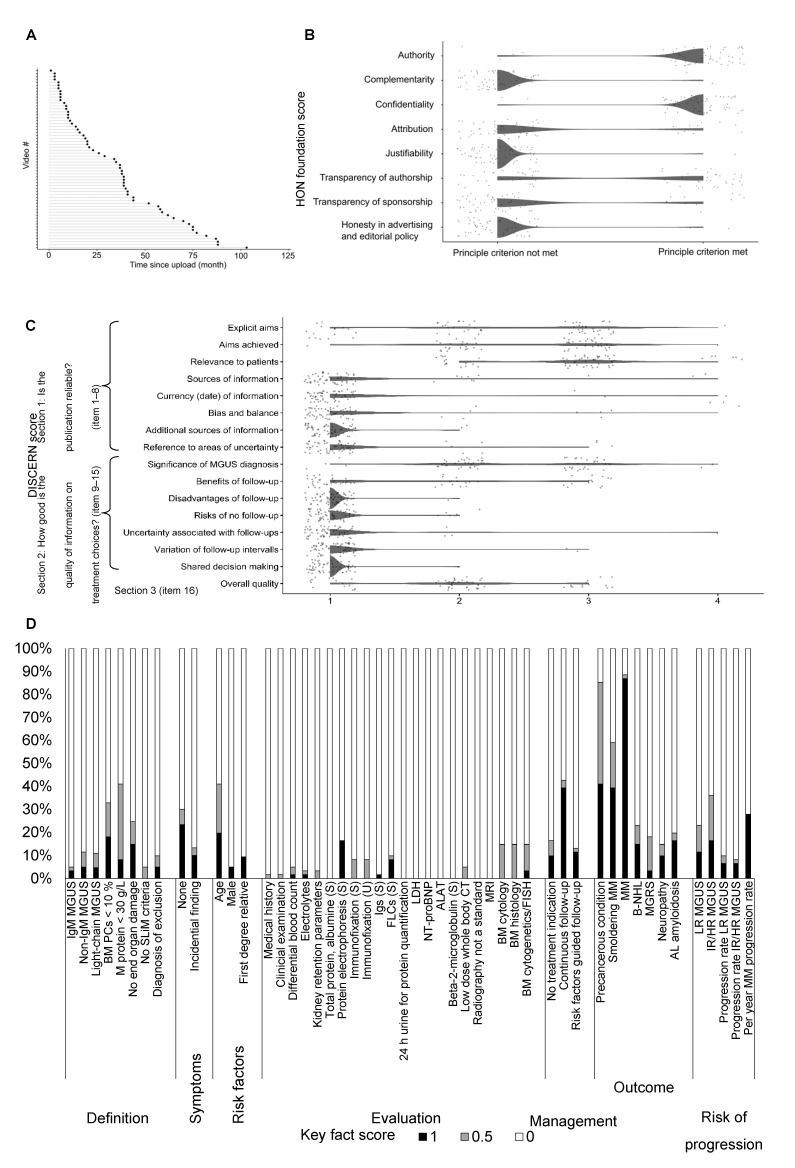
Characterization of unique videos. (**A**) The lollipop plot shows the videos ordered by their time since upload until the date of assessment in months. (**B**) Scatter dot plot shows the score result reached by every single video (*n* = 61) for each item of the HON Foundation score (0, principle criterium not met; 1, principle criterium met). To avoid a visual overlap the dots were spread around the respective score category. (**C**) Scatter dot plot shows the score result reached by every single video (*n* = 61) for each item of the DISCERN score. The categorial item scoring ranges between 1 (not addressed/fulfilled) and 5 (fully addressed/fulfilled). To avoid a visual overlap, the dots were spread around the respective score category. (**D**) For each of the 50 key fact items, the proportion of videos (*n* = 61) fully (1), partially (0.5) or not (0) addressing the respective contents is shown. The results are grouped by key fact category.

Most videos were provided by news/media (*n* = 32, 52.5%), followed by foundations/advocacies (*n* = 21, 34.4%). Only a few videos were provided by a scientific/governmental organization (*n* = 2, 3.3%). Most videos originated from North America (*n* = 51, 83.6%). The median time since upload of the evaluated videos was 34 months (Figure 2A). In median, the videos had a duration of 4 (range 1–57) minutes and 452 (range 24–55,869) views. A median viewing rate of 0.81 (range 0.02–28.16) indicated that in median the videos were watched less than once a day. The engagement rate was very low (median 0.01, range 0.00–0.05), resulting from low numbers of likes, dislikes, and comments.

As the JAMA score and HON Foundation certificate refer to websites, the general quality of medical information online was assessed in a step-by-step evaluation of videos according to the eight HON Foundation principle criteria. Of 8 maximum points, in median 3 (range 1–6) points were achieved by the evaluated videos. While the items authority, confidentiality, and transparency of authorship were addressed by the majority of videos, aspects of complementarity, attribution, justifiability, transparency of sponsorship, honesty in advertising, and editorial policy were generally not met (Figure 2B). Overall, only one video (1.6%) reached a high quality according to the HON Foundation score. Thiry-five videos (57.4%) were ranked as medium and 25 videos (40.1%) as low quality according to the HON Foundation score.

The patient- (user-) focused quality of medical information online assessed by the DISCERN score was rather poor: of 16 minimum and 80 maximum possible points, in median 24 (range 18–35) points were achieved by the evaluated videos. The items “explicit aims”, “aims achieved”, “relevance to patients” and “significance of MGUS diagnosis” were at least addressed in most videos. However, the remaining items, including the most items on the quality of information on treatment/follow-up choices (section 2) were provided by very few videos (Figure 2C, Figure S3B, Table S4).

The MGUS-related content was evaluated by a set of 50 very detailed key facts (Figure 2D, Figure S3C). Of 50 maximum possible points, in median 6 (range 0–22) points were achieved, demonstrating a low informational content. The content addressed in most videos related to a possible outcome of the precancerous condition MGUS, i.e., transition in SM and/or MM. Aspects of MGUS diagnosis criteria, absence of symptoms, risk factors, management, and risk of progression were content in not more than one-third of the evaluated videos. The details on the evaluation of MGUS were addressed only in a few videos. Eight videos (13.1%) containing an overall 25 misleading/wrong facts were identified (Table 3). Twenty misleading/wrong facts (80.0%) related to the definition of MGUS diagnosis: MGUS was falsely described to be associated with symptoms of end-organ damage—which contradicts the diagnosis criteria of MGUS. The misleading/wrong facts on risk factors (*n* = 2, 8.0%) established a causal link between MGUS and environmental toxins or viral infection, which is not proven so far. Moreover, regarding the management (*n* = 2, 8.0%) steroid treatment and regular bone density assessment were suggested, which is not recommended in the current guidelines.

A data set containing the original data on selected quality, content scores, and citations of misleading/wrong facts obtained from the unique videos is provided along with this manuscript (Table S6).

#### 3.2.3. Video Characteristics According to Category

In order to identify differences, a comparison of videos by providers was performed (foundation/advocacy *n* = 21, news/media *n* = 32, Table 5). Due to the low number, videos provided by scientific/governmental institutions (*n* = 2), industry/for-profit organizations (*n* = 2), and personal commentary/blogs (*n* = 4) were not included.

Videos provided by foundations/advocacies had a significantly higher number of views (median 885, range 24–55,869) compared to news/media (median 207, range 26–6245), (*p* = 0.004). This resulted in a higher viewing rate (median 1.03 versus 053). However, the viewing rate difference was statistically not significant (*p* = 0.075).

MGUS videos provided by foundations/advocacies provided a higher general quality of medical information online judged by the HON Foundation score. In the first-mentioned category the most videos (*n* = 18, 85.7%) achieved a medium HON quality rank, while in the latter category only 12 videos (37.5%) achieved this rank and other videos were ranked as low (*n* = 20, 62.5%, *p* < 0.001, Figure S3A).

No statistically significant differences were identified regarding the patient- (user-) focused quality of medical information online according to the DISCERN score (*p* = 0.857, Figure S3B). There was also no difference in the MGUS key facts addressed in videos of both categories (*p* = 0.368, Figure S3C).

### 3.3. Comparison between Websites and Videos on MGUS

A comparison of unique websites (*n* = 86) and videos (*n* = 61) was possible, in some, but not all assessed variables (Table 2).

While scientific/governmental institutions (*n* = 38, 44.2%) and foundations/advocacies (*n* = 31, 36.1%) provided the most websites, the videos were mainly provided by news/media (*n* = 32, 52.5%) and foundations/advocacies (*n* = 21, 34.4%), (*p* < 0.001).

The patient- (user-) focused quality of medical information online evaluated by the DISCERN score (minimum 16, maximum 80 points) was poor in both websites (median 27, range 16–43) and videos (median 24, range 18–35), with no statistically significant difference (*p* = 0.246).

The amount of MGUS-related content evaluated by the sum key fact score (minimum 0, maximum 50 points) provided by websites (median 13, range 3–37) was higher compared to videos (median 6, range 0–22), (*p* < 0.001).

## 4. Discussion

The current analysis provides an extensive evaluation of MGUS-related online health information. Previously published analyses on the reliability and accuracy of online health information mainly focus on otorhinolaryngology-related topics [23,24,25,26,27,28,29,30]. Studies on other medical conditions, such as idiopathic pulmonary fibrosis, SARS-CoV-2, neurological disorders, etc., are available as well [15,31,32,33,34,35,36]. However, except for oral precancerous conditions, other precancerous and cancer entities were not in the focus of such evaluations [37,38]. As associated with a constant fear of facing cancer, precancerous conditions might be, in particular, the focus of a patients’ online search and the quality of available online information is crucial.

Besides its novelty, our study shows methodological strengths. A significant number of MGUS-related websites was analyzed upon a broad online search of three search engines. Going beyond written content, videos were considered. As Google and YouTube are the two most visited search platforms, the selection of analyzed websites and videos might be considered representative [39]. The evaluation was performed in a reproducible and objective score-based manner, applying a set of well-established scores covering the aspects of general, patient- (user-) focused quality, and MGUS-related content.

The limitations of the study can mainly be attributed to constantly varying and potentially growing numbers, continuous updating, and changes in the rating of websites and videos. Therefore, the website and video selection in this analysis cannot be considered as ultimate.

The website search on Google, Bing, and Yahoo revealed a lack of integrity/insufficient search results which was reflected by a high number of websites with no relevance to MGUS. The median time since last website update was 24 months, which reflects an outdated informational status. The actuality of health online information is of outstanding importance and should be critically considered [40]. Of importance, the website search rank in each search engine does not necessarily correlate with quality [41]. Indeed, in the current analysis, the most qualitative websites were not ranked at a top position. Moreover, the websites did not differ in terms of analyzed scores between the three search engines or regarding their search rank. Thus, not only the search engine market leader Google but also other search engines might be used, which is advantageous for users [39].

The 86 unique MGUS-relevant websites showed a medium general quality of medical information online and less than 10% of websites had a valid HON Foundation certificate. This is in line with the facts—only a small proportion of medical and health websites provide quality scores and certificates, and identification of websites with high-quality information is challenging for patients [42]. The patient-focused quality of MGUS-related information was poor, with, therefore, questionable potential to reduce anxiety and concerns and support the compliance for regular follow-up examinations. The overall reading level required at least an 11th US school grade. As “most adults read at an 8th grade level, and 20% of the population reads at or below a 5th grade level”, the content would have been difficult to understand for the vast majority of patients [43]. The informational content level was very low. As we applied a large set of stringent criteria based on current diagnostic and clinical guidelines, a high sum content key fact score was not expected [1,2,22]. However, 12.8% of websites contained misleading/wrong facts, holding the risk of misinformation. Websites provided by scientific/governmental organizations were similar to those provided by foundations/advocacies or news/media, in terms of all analyzed variables except a slightly higher content level. Overall, our results are in line with these previously published studies. Those conclude on frequently outdated, mixed-, or low-quality, and incomplete online health information that requires high readability skills [15,23,24,25,26,27,28,29,30,31,32,33,34,35,36,37,38].

Given a median time since video upload of 34 months, the MGUS-related video content can be considered as outdated. Judged by the HON Foundation score, the general quality of medical information online can be considered as medium. The patient- (user-) focused quality of MGUS-related information was poor and therefore similar to MGUS-related websites. The content level was very low, even significantly lower compared to websites. MGUS videos provided by foundations/advocacies provided a higher general quality of medical information online judged by the HON Foundation score. To the best of our knowledge, this is the first systematical and score-based evaluation of disease-related videos that goes beyond the assessment of provided medical content. We could identify only one “snapshot analysis of information available on youtube.com” published by Tan et al., that evaluates the quality of content regarding breast reconstruction in breast cancer patients [44]. Similar to the results obtained in the current study, the authors conclude that the YouTube videos do not provide comprehensive information.

## 5. Conclusions

The current analysis of online information on MGUS revealed an overall low general, patient- (user-) focused and content quality provided on a high reading level. Thus, understandability and informative value can be attributed to single sources only. Therefore, regular review, incorporation of indices indicating the quality of provided online health information, and consideration of current MGUS guidelines is warranted.

## Figures and Tables

**Table 1:** Search results for websites and videos.

Health Information Source	A. Websites			B. Videos
Search Engine/Platform	Google	Bing	Yahoo	YouTube
**Initial search with three search terms, *n* (%)**	300 (100)	300 (100)	300 (100)	150 (100)
**Duplicates between the three search terms, *n* (%)**	176 (58.7)	185 (61.7)	201 (67.0)	62 (41.3)
**Eligibility criteria not met, *n***				
No relevance to MGUS	38	32	24	19
Access required	9	5	6	1
Other language	5	2	0	1
Scientific article	19	10	10	0
Duplicate	/	/	/	6
∑ (%)	71 (23.7)	49 (16.3)	40 (13.3)	27 (18.0)
**Included websites for search engine, *n* (%)**	53 (17.7)	66 (22.0)	59 (19.7)	/
**Overlap, *n***				
Google/Bing	37			/
Bing/Yahoo	55			/
Yahoo/Google	34			/
Google/Bing/Yahoo	30			/
**Overall unique, *n* (%)**	**86 (9.5)**			**61 (40.7)**

MGUS, monoclonal gammopathy of undetermined significance.

**Table 2:** Characterization of unique websites and videos.

Health Information Source	A. Websites	B. Videos	*p*-Value
Overall unique websites/videos, *n* (%)	86 (100)	61 (100)	/
**Website/video category, *n* (%)**			
Scientific/governmental	38 (44.2)	2 (3.3)	<0.001
Foundation/advocacy	31 (36.1)	21 (34.4)	
News/media	13 (15.1)	32 (52.5)	
Industry/for profit	2 (2.3)	2 (3.3)	
Personal commentary/blog	2 (2.3)	4 (6.6)	
**Host continent, *n* (%)**			
Europe	11 (12.8)	9 (14.8)	0.858
North America	68 (79.1)	51 (83.6)	
South America	1 (1.2)	0 (0.0)	
Asia	1 (1.2)	0 (0.0)	
Australia	3 (3.5)	0 (0.0)	
Africa	0 (0.0)	0 (0.0)	
Antarctica	0 (0.0)	0 (0.0)	
Not assessable	2 (2.3)	1 (1.6)	
**HON Foundation certificate/score**			
Assessable, *n* (%)	86 (100)	61 (100)	
Not assessable, *n* (%)	0 (0.0)	0 (0.0)	
Valid certificate, *n* (%)	7 (8.1)	/	/
Median (range)	/	3 (1–6)	/
Top 10 websites/videos, valid certificate (*n*) or median (range)	1	4 (3–5)	/
Rating according to HON Foundation score, *n* (%)			
Low	/	25 (40.1)	/
Medium	/	35 (57.4)	
High	/	1 (1.6)	
**JAMA score**			
Assessable, *n* (%)	86 (100)	/	
Not assessable, *n* (%)	0 (0.0)	/	
Median (range)	3 (0–4)	/	/
**Flesch Reading Ease score**			
Assessable, *n* (%)	76 (88.4)	/	
Not assessable, *n* (%)	10 (11.6)	/	
Mean (SD)	48 (10)	/	/
**Flesch Kincaid Grade Level**			
Assessable, *n* (%)	76 (88.4)	/	
Not assessable, *n* (%)	10 (11.6)	/	
Mean (SD)	11 (3)	/	/
**Video duration, minutes**			
Median (range)	/	4 (1–57)	/
Mean (SD)	/	7 (9)	
**Views, median (range)**	/	452 (24–55,869)	/
**Likes, median (range)**	/	5 (0–385)	/
**Dislikes, median (range)**	/	0 (0–14)	/
**Comments, median (range)**	/	0 (0–69)	/
**Viewing rate, median (range)**	/	0.81 (0.02–28.16)	/
**Engagement rate, median (range)**	/	0.01 (0.00–0.05)	
**Sum DISCERN score**			
Assessable, *n* (%)	84 (97.7)	61 (100)	
Not assessable, *n* (%)	2 (2.3)	0 (0.0)	
Median (range)	27 (16–43)	24 (18–35)	0.246
Top 10 websites/videos, median (range)	32 (20–43)	21 (19–33)	/
**Sum key fact score**			
Assessable, *n* (%)	86 (100)	61 (100)	
Not assessable, *n* (%)	0 (0.0)	0 (0.0)	
Median (range)	13 (3–37)	6 (0–22)	<0.001
Top 10 websites/videos, median (range)	17 (6–37)	16 (5–18)	/
**Misleading/wrong facts**			
Websites/videos with misleading/wrong facts, *n* (%)	11 (12.8)	8 (13.1)	/
Overall identified wrong facts, *n*	40	25	/

HON, Health on the Net; JAMA, Journal of the American Medical Association; SD, standard deviation.

**Table 3:** Misleading and wrong facts on websites and videos.

Health Information Source	A. Websites	B. Videos
**Overall unique websites/videos, *n* (%)**	86 (100)	61 (100)
**Websites/videos with misleading/wrong facts, *n* (%)**	11 (12.8)	8 (13.1)
By website category, *n*		
Scientific/governmental	2	0
Foundation/advocacy	5	2
News/media	3	3
Industry/for-profit	1	0
Personal commentary/blog	0	3
**Misleading/wrong facts**		
Overall, *n*	40	25
By key fact category, *n* (%)		
Definition	10 (25.0)	20 (80.0)
Symptoms	0 (0.0)	0 (0.0)
Risk factors	9 (22.5)	2 (8.0)
Evaluation	3 (7.5)	0 (0.0)
Management	15 (37.5)	2 (8.0)
Outcome	3 (7.5)	0 (0.0)
Risk of progression	0 (0.0)	1 (4.0)

**Table 4:** Characterization of websites by category.

Website Category	Scientific/Governmental	Foundation/Advocacy	News/Media	*p*-Value
**Websites, *n***	38	31	13	
**Host continent, *n* (%)**				
Europe	3 (7.9)	6 (19.4)	1 (7.7)	0.282 ^a^
North America	32 (84.2)	22 (71.0)	11 (84.6)	
South America	1 (2.6)	0 (0.0)	0 (0.0)	
Asia	0 (0.0)	0 (0.0)	1 (7.7)	
Australia	2 (5.3)	1 (3.2)	0 (0.0)	
Africa	0 (0.0)	0 (0.0)	0 (0.0)	
Antarctica	0 (0.0)	0 (0.0)	0 (0.0)	
Not assessable	0 (0.0)	0 (0.0)	0 (0.0)	
**HON Foundation certificate**				
Assessable, *n* (%)	38 (100)	31 (100)	13 (100)	
Not assessable, *n* (%)	0 (0.0)	0 (0.0)	0 (0.0)	
Valid certificate, *n* (%)	3 (7.9)	3 (9.7)	1 (7.7)	0.959
**JAMA score**				
Assessable, *n* (%)	38 (100)	31 (100)	13 (100)	
Not assessable, *n* (%)	0 (0.0)	0 (0.0)	0 (0.0)	
Median (range)	3 (1–4)	3 (0–4)	3 (1–4)	0.926
**Flesch Reading Ease score**				
Assessable, *n* (%)	30 (78.9)	29 (93.5)	13 (100)	
Not assessable, *n* (%)	8 (21.1)	2 (6.5)	0 (0.0)	
Mean (SD)	48 (9)	47 (10)	50 (13)	0.539
**Flesch Kincaid Grade level**				
Assessable, *n* (%)	27 (71.1)	28 (90.3)	10 (76.9)	
Not assessable, *n* (%)	11 (28.9)	3 (9.7)	3 (23.1)	
Mean (SD)	11 (2)	11 (2)	11 (3)	0.939
**Sum DISCERN score**				
Assessable, *n* (%)	38 (100)	29 (93.5)	13 (100)	
Not assessable, *n* (%)	0 (0.0)	2 (6.5)	0 (0.0)	
Median (range)	28 (19–43)	27 (16–38)	23 (17–42)	0.119
**Sum key fact score**				
Assessable, *n* (%)	38 (100)	31 (100)	13 (100)	
Not assessable, *n* (%)	0 (0.0)	0 (0.0)	0 (0.0)	
Median (range)	17 (4–37)	11 (5–35)	11 (5–33)	0.022
**Misleading/wrong facts**				
Websites with misleading/wrong facts, *n* (%)	2 (5.3)	5 (16.1)	3 (23.1)	0.143
Overall identified wrong facts, *n*	11	17	11	

^a^ Europe versus North America. HON, Health on the Net; JAMA, Journal of the American Medical Association; SD, standard deviation.

**Table 5:** Characterization of videos by category.

Group	Foundation/Advocacy	News/Media	*p*-Value
**Video category, *n***	21	32	
**Host continent, *n* (%)**			
Europe	2 (9.5)	7 (21.9)	/
North America	19 (90.5)	25 (78.1)	
South America	0 (0.0)	0 (0.0)	
Asia	0 (0.0)	0 (0.0)	
Australia	0 (0.0)	0 (0.0)	
Africa	0 (0.0)	0 (0.0)	
Antarctica	0 (0.0)	0 (0.0)	
Not assessable	0 (0.0)	0 (0.0)	
**Video duration, minutes**			
Median (range)	4 (1–57)	2 (1–20)	
Mean (SD)	8 (13)	5 (5)	0.246
**Views, median (range)**	885 (24–55,869)	207 (26–6245)	0.004
**Likes, median (range)**	6 (0–385)	4 (0–65)	0.080
**Dislikes, median (range)**	0 (0–14)	0 (0–5)	0.390
**Comments, median (range)**	0 (0–33)	0 (0–2)	0.084
**Viewing rate, median (range)**	1.03 (0.02–28.16)	0.53 (0.12–28.16)	0.075
**Engagement rate, median (range)**	0.01 (0.00–0.04)	0.01 (0.00–0.04)	0.348
**HON Foundation score**			
Assessable, *n* (%)	21 (100)	32 (100)	
Not assessable, *n* (%)	0 (0.0)	0 (0.0)	
Median (range)	3 (2–6)	2 (2–5)	0.007
Rating according to HON Foundation score, *n* (%)			
Low	2 (9.5)	20 (62.5)	<0.001
Medium	18 (85.7)	12 (37.5)	
High	1 (4.8)	0 (0.0)	
**Sum DISCERN score**			
Assessable, *n* (%)	21 (100)	32 (100)	
Not assessable, *n* (%)	0 (0.0)	0 (0.0)	
Median (range)	2 (1–3)	2 (1–3)	0.857
**Sum key fact score**			
Assessable, *n* (%)	21 (100)	32 (100)	
Not assessable, *n* (%)	0 (0.0)	0 (0.0)	
Median (range)	6 (2–22)	5 (0–17)	0.368
**Misleading/wrong facts**			
Videos with misleading/wrong facts, *n* (%)	2 (10.0)	3 (9.4)	/
Overall identified wrong facts, *n*	9	4	/

HON, Health on the Net; SD, standard deviation.

## Data Availability

Data supporting reported results are provided along with the manuscript (Tables S5 and S6).

## Supplementary Materials

The following are available online at https://www.mdpi.com/article/10.3390/cancers13184508/s1, Figure S1: Characterization of websites by search rank and time since last update, Figure S2: Characterization of websites by category, Figure S3: Characterization of videos by category, Table S1: Scores, measures and certificates used for the evaluation of websites and videos, Table S2: Website and video content evaluation by MGUS key facts, Table S3: Characterization of websites by search engine, Table S4: DISCERN score for websites and videos, Table S5: Website data, Table S6: Video data.
