# Supplementary material for: Accuracy and Reliability of Internet Resources for Information on Monoclonal Gammopathy of Undetermined Significance—What Information Is out There for Our Patients?
Source: Cancers (Basel). 2021 Sep 7;13(18):4508. doi: 10.3390/cancers13184508 (PMC8465467; doi:10.3390/cancers13184508)
Supplement: Supplementary file 1 [file cancers-13-04508-s001.zip › cancers-1361372-supplementary/Supplementary Material/Figure S1.pptx]

## Slide 1
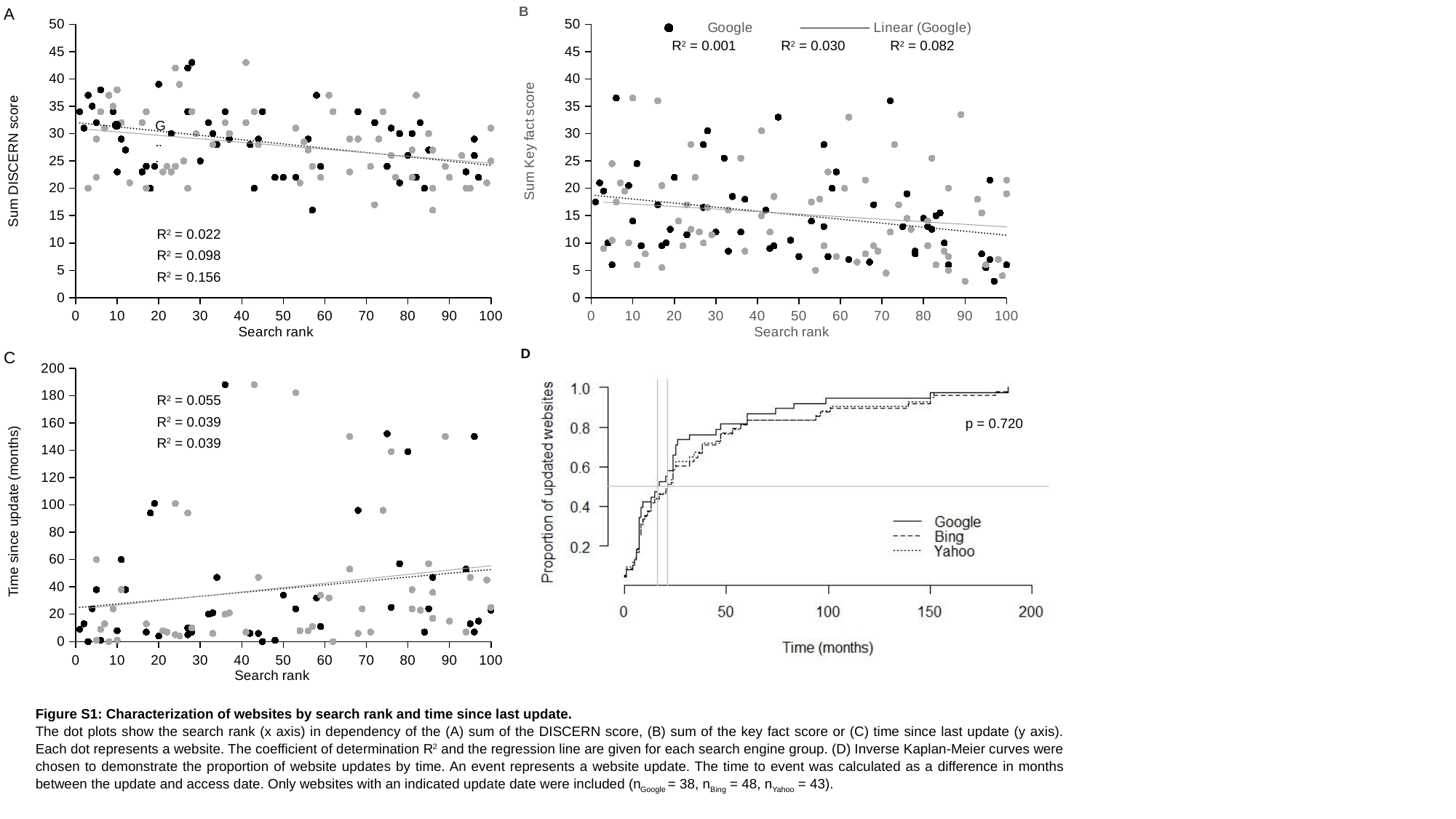

### Chart: A
| Category | | | |
|---|---|---|---|
### Chart: B
| Category | | | |
|---|---|---|---|R2 = 0.001	R2 = 0.030	R2 = 0.082
R2 = 0.022
R2 = 0.098
R2 = 0.156
D
### Chart: C
| Category | | | |
|---|---|---|---|
R2 = 0.055
R2 = 0.039
R2 = 0.039
p = 0.720
Figure S1: Characterization of websites by search rank and time since last update.
The dot plots show the search rank (x axis) in dependency of the (A) sum of the DISCERN score, (B) sum of the key fact score or (C) time since last update (y axis). Each dot represents a website. The coefficient of determination R2 and the regression line are given for each search engine group. (D) Inverse Kaplan-Meier curves were chosen to demonstrate the proportion of website updates by time. An event represents a website update. The time to event was calculated as a difference in months between the update and access date. Only websites with an indicated update date were included (nGoogle = 38, nBing = 48, nYahoo = 43).
