# Supplementary material for: Accuracy and Reliability of Internet Resources for Information on Monoclonal Gammopathy of Undetermined Significance—What Information Is out There for Our Patients?
Source: Cancers (Basel). 2021 Sep 7;13(18):4508. doi: 10.3390/cancers13184508 (PMC8465467; doi:10.3390/cancers13184508)
Supplement: Supplementary file 1 [file cancers-13-04508-s001.zip › cancers-1361372-supplementary/Supplementary Material/Figure S2.pptx]

## Slide 1
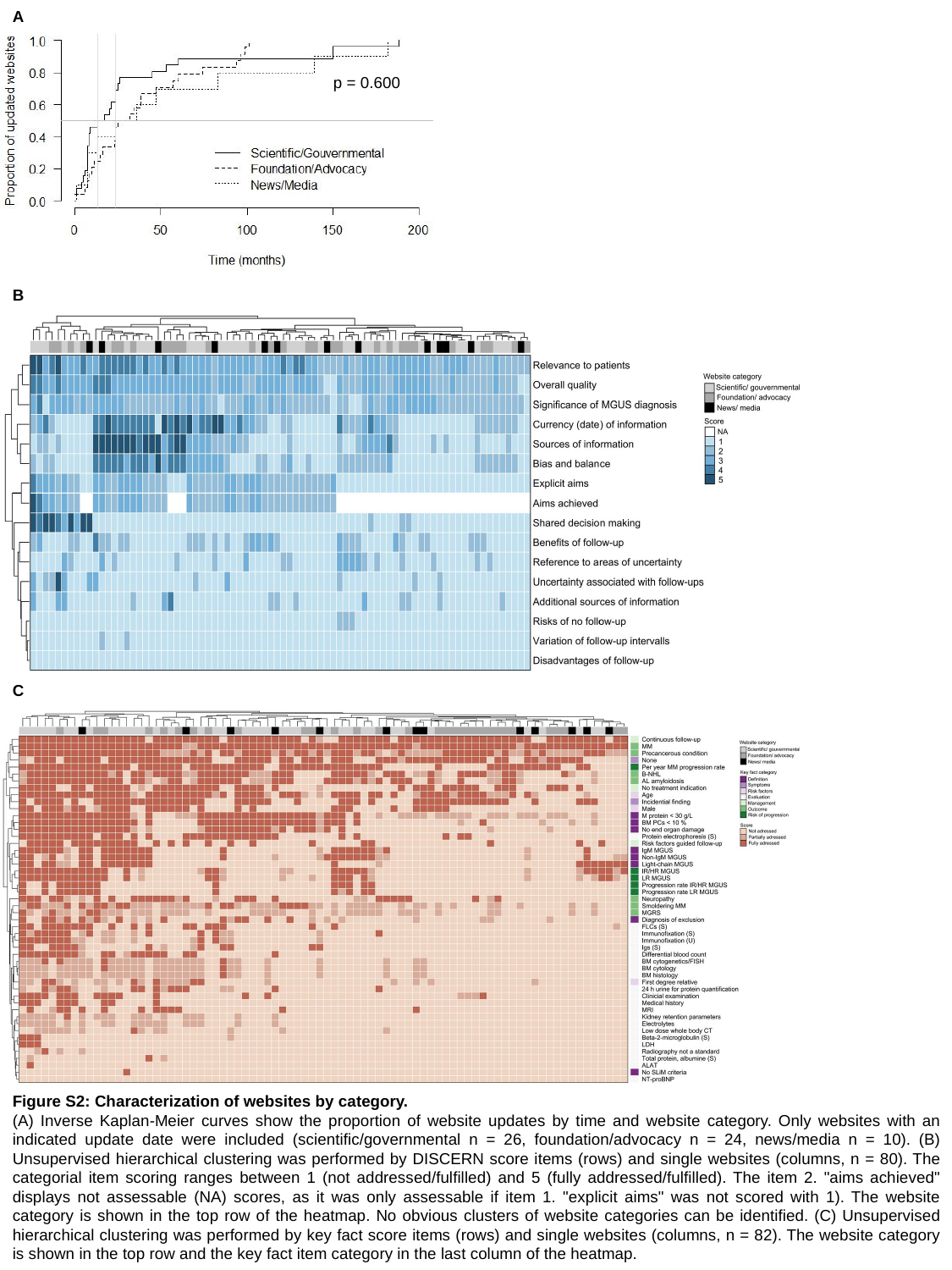

A
p = 0.600
B
C
Figure S2: Characterization of websites by category.
(A) Inverse Kaplan-Meier curves show the proportion of website updates by time and website category. Only websites with an indicated update date were included (scientific/governmental n = 26, foundation/advocacy n = 24, news/media n = 10). (B) Unsupervised hierarchical clustering was performed by DISCERN score items (rows) and single websites (columns, n = 80). The categorial item scoring ranges between 1 (not addressed/fulfilled) and 5 (fully addressed/fulfilled). The item 2. "aims achieved" displays not assessable (NA) scores, as it was only assessable if item 1. "explicit aims" was not scored with 1). The website category is shown in the top row of the heatmap. No obvious clusters of website categories can be identified. (C) Unsupervised hierarchical clustering was performed by key fact score items (rows) and single websites (columns, n = 82). The website category is shown in the top row and the key fact item category in the last column of the heatmap.
