# Supplementary material for: Accuracy and Reliability of Internet Resources for Information on Monoclonal Gammopathy of Undetermined Significance—What Information Is out There for Our Patients?
Source: Cancers (Basel). 2021 Sep 7;13(18):4508. doi: 10.3390/cancers13184508 (PMC8465467; doi:10.3390/cancers13184508)
Supplement: Supplementary file 1 [file cancers-13-04508-s001.zip › cancers-1361372-supplementary/Supplementary Material/Figure S3.pptx]

## Slide 1
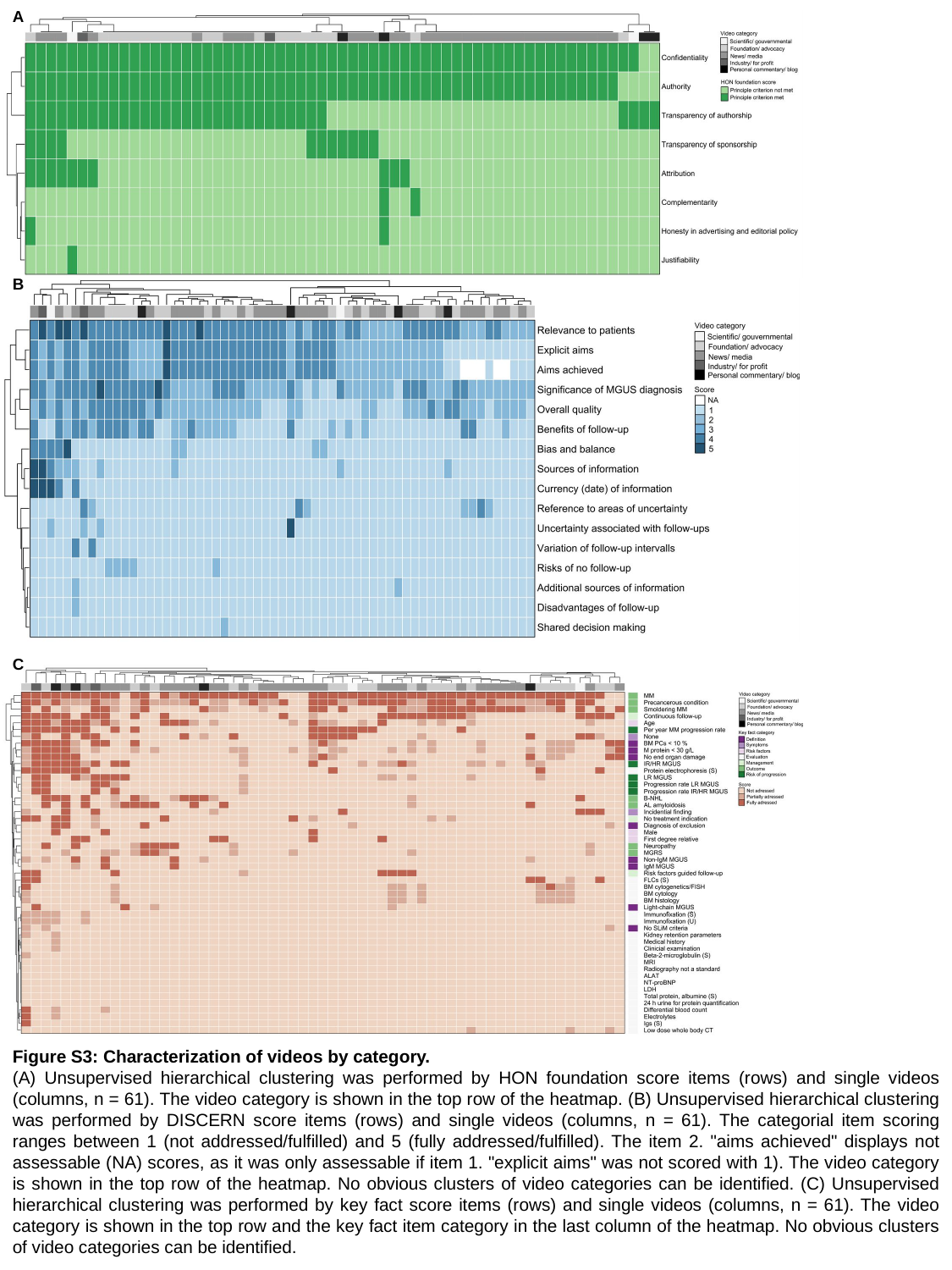

A
B
C
Figure S3: Characterization of videos by category.
(A) Unsupervised hierarchical clustering was performed by HON foundation score items (rows) and single videos (columns, n = 61). The video category is shown in the top row of the heatmap. (B) Unsupervised hierarchical clustering was performed by DISCERN score items (rows) and single videos (columns, n = 61). The categorial item scoring ranges between 1 (not addressed/fulfilled) and 5 (fully addressed/fulfilled). The item 2. "aims achieved" displays not assessable (NA) scores, as it was only assessable if item 1. "explicit aims" was not scored with 1). The video category is shown in the top row of the heatmap. No obvious clusters of video categories can be identified. (C) Unsupervised hierarchical clustering was performed by key fact score items (rows) and single videos (columns, n = 61). The video category is shown in the top row and the key fact item category in the last column of the heatmap. No obvious clusters of video categories can be identified.
