# Supplementary material for: Accuracy and Reliability of Internet Resources for Information on Monoclonal Gammopathy of Undetermined Significance—What Information Is out There for Our Patients?
Source: Cancers (Basel). 2021 Sep 7;13(18):4508. doi: 10.3390/cancers13184508 (PMC8465467; doi:10.3390/cancers13184508)
Supplement: Supplementary file 1 [file cancers-13-04508-s001.zip › cancers-1361372-supplementary/Supplementary Material/Table S2.pdf]

Table S2: Website and video content evaluation by MGUS key facts.

Category	Item	Description
**Definition**	IgM MGUS	Serum IgM M protein < 30 g/L, BM lymphoplasmacytic infiltration < 10 %, no symptoms or end-organ damage that can be attributed to the lymphoproliferative disorder.
	Non-IgM MGUS	Serum M protein (non-IgM type) < 30 g/L, clonal BM PCs < 10 %, absence of end-organ damage.
	Light-chain MGUS	Abnormal FLC ratio, increased level of the involved LC, no heavy chain expression on immunofixation, absence of end-organ damage, clonal BM PCs <10 %, urinary M protein < 500 mg/24 h.
	BM PCs < 10 %	self-explanatory
	M protein < 30 g/L	self-explanatory
	No end organ damage	No evidence of CRAB criteria, i.e. hyper**c**alcemia, **r**enal insufficiency, **a**nemia, **b**one lesions.
	No SLiM criteria	SLiM criteria: clonal BM PCs < **s**ixty percent, involved:uninvolved serum FLC ratio < 100, ≤ 1 focal lesions on MRI studies.
	Diagnosis of exclusion	No diagnostic criteria of other hemato-oncological entities met.
**Symptoms**	None	No evidence of symptoms that can be attributed to MGUS.
	Incidental finding	MGUS is often diagnosed incidentally.
**Risk factors**	Age	Incidence increases with age.
	Male	Slightly higher incidence in men compared to women.
	First degree relative	Increased risk in case of first-degree relatives diagnosed with MGUS.
**Evaluation**	Medical history	Mandatory to obtain any relevant information on patient's health status.
	Clinical examination	Mandatory to assess any signs or symptoms of a medical condition.
	Differential blood count	Differentiation of white blood cells.
	Electrolytes	Sodium, potassium, calcium etc.
	Kidney retention parameters	Creatinine, glomerular filtration rate, urea.
	Total protein, albumin (S)	Quantity of total protein and albumin in serum.
	Protein electrophoresis (S)	Separates the serum protein components into five major fractions. Examines which globulin fraction is elevated.
	Immunofixation (S)	Assessment of the M protein type in serum.
	Immunofixation (U)	Assessment of the M protein type in urine.
	Ig (S)	Concentration of IgG, IgA, IgM in serum.
	FLCs (S)	Concentration of FLCs (kappa and lambda) in serum.
	24 h urine for protein quantification	Quantifies the urinary protein excretion per 24 h.
	LDH	Non-specific marker of cell turnover.
	NT-proBNP	Normal level helps to rule out chronic heart failure (particularly relevant in case of AL amyloidosis).
	ALAT	Evaluation of liver function.
	Beta-2-microglobulin (S)	(Prognostic) marker in PC disorders, particularly MM.
	Low dose whole body CT	Detection of osteolyses and osteopenia, without contrast agents.
	Radiography not a standard	Projection radiography is no longer standard of diagnostics in PC disorders.
	MRI	Evaluation of diffuse BM infiltration, focal bone lesions and extramedullary manifestations.
	BM cytology	Assessment of extent of PC infiltration and status of other hematopoietic cells.
	BM histology	Assessment of extent of PC infiltration and status of other hematopoietic cells in the context of BM stroma.
	BM cytogenetics/FISH	Detection of chromosomal aberrations.
**Management**	No treatment indication	No treatment indication is given when MGUS diagnosis is established and differential diagnoses or organ damage are excluded.
	Continuous follow-up	Continuous follow-up is mandatory to detect disease progression or organ damage and initiate treatment.
	Risk factors guided follow-up	Follow-up intervals might be defined in dependency of presence/absence of risk factors.

**Outcome**	Precancerous condition	MGUS can progress into a hemato-oncological disorder and end organ damage might develop, i.e. smoldering MM, MM, B-NHL, MGRS, neuropathy, AL amyloidosis.
	Smoldering MM	Serum M protein ≥ 30 g/L or urinary M protein ≥ 500 mg/24 h or BM PCs ≥ 10-60 %, absence of MM defining events or AL amyloidosis.
	MM	Clonal BM PCs ≥ 10% or biopsy proven plasmacytoma and at least one of the CRAB or SLiM criteria.
	B-NHL	Particularly in case of IgM-MGUS is B-NHL the underlying disease that might require treatment in case of progression.
	MGRS	Monoclonal gammopathy with unclear renal insufficiency or significant proteinuria. Criteria of multiple myeloma or other lymphoproliferative diseases not met.
	Neuropathy	Deposits of non-functional M protein at peripheral nerves can cause neuropathy.
	AL amyloidosis	Deposits of non-functional M protein can affect nearly every organ and cause severe organ damage.
**Risk of progression**	LR MGUS	Serum M protein < 15 g/L, normal FLC ratio.
	IR/HR MGUS	All other than LR MGUS.
	Progression rate LR MGUS	Approximately 5 % in 20 years.
	Progression rate IR/HR MGUS	20-60 % in 20 years.
	Per year MM progression rate	The risk to develop a MM or another lymphoproliferative disorder is approximately 1 % per year.

MGUS key facts were sourced from Blood guideline "How I manage monoclonal gammopathy of undetermined significance", International Myeloma Working Group updated criteria for the diagnosis of multiple myeloma [and MGUS]. and Deutsche Gesellschaft für Hämatologie und Medizinische Onkologie e. V. (DGHO) on MGUS.[1-3]
AL-amyloidosis, light chain-amyloidosis; ALAT, alanine aminotransferase; BM, bone marrow; B-NHL, B-cell non-Hodgkin lymphoma; CT, computer tomography; FLC, free light chain; FISH, fluorescence in-situ hybridization; HR, high-risk; Ig, immunoglobulin; IR, intermediate-risk; LC, light chain; LDH, lactate dehydrogenase; LR, low-risk; MGRS, monoclonal gammopathy of renal significance; MGUS, monoclonal gammopathy of undetermined significance; MM, multiple myeloma; M protein, monoclonal protein; MRI, magnetic resonance imaging; NT-proBNP, N-terminal pro b-type natriuretic peptide; PC, plasma cell; S, serum; U, urine.

1. Go RS, Rajkumar SV. How I manage monoclonal gammopathy of undetermined significance. *Blood* 2018; **131**(2)**:** 163-173. e-pub ahead of print 2017/12/01; doi: 10.1182/blood-2017-09-807560

2. Rajkumar SV, Dimopoulos MA, Palumbo A, Blade J, Merlini G, Mateos MV *et al.* International Myeloma Working Group updated criteria for the diagnosis of multiple myeloma. *Lancet Oncol* 2014; **15**(12)**:** e538-548. doi: 10.1016/S1470-2045(14)70442-5

3. Scheid C, Driessen C, Knop S, Krauth MT, Naumann R, Schieferdecker A *et al.* Monoklonale Gammopathie unklarer Signifikanz (MGUS). In: DGHO, 2019.
