# Supplementary material for: Accuracy and Reliability of Internet Resources for Information on Monoclonal Gammopathy of Undetermined Significance—What Information Is out There for Our Patients?
Source: Cancers (Basel). 2021 Sep 7;13(18):4508. doi: 10.3390/cancers13184508 (PMC8465467; doi:10.3390/cancers13184508)
Supplement: Supplementary file 1 [file cancers-13-04508-s001.zip › cancers-1361372-supplementary/Supplementary Material/Table S3.pdf]

Table S3: Characterization of websites by search engine.

Search engine	Google	Bing	Yahoo	*P* value
**Websites, n**	53	66	59	
**Website category, n (%)**				
Scientific/governmental	24 (45.3)	30 (45.5)	29 (49.2)	0.096[a]
Foundation/advocacy	15 (28.3)	27 (40.9)	25 (42.4)	
News/media	12 (22.6)	5 (7.6)	3 (5.1)	
Industry/for profit	2 (3.8)	2 (3.0)	2 (3.4)	
Personal commentary/blog	0 (0.0)	2 (3.0)	0 (0.0)	
**Host continent, n (%)**				
Europe	7 (13.2)	7 (10.6)	7 (11.9)	0.937[b]
North America	43 (81.1)	53 (80.3)	47 (79.7)	
South America	1 (1.9)	0 (0.0)	0 (0.0)	
Asia	0 (0.0)	1 (1.5)	0 (0.0)	
Australia	2 (3.8)	3 (4.5)	3 (5.1)	
Africa	0 (0.0)	0 (0.0)	0 (0.0)	
Antarctica	0 (0.0)	0 (0.0)	0 (0.0)	
Not assessable	0 (0.0)	2 (3.0)	2 (3.4)	
**HON foundation certificate**				
Assessable, n (%)	53 (100)	66 (100)	59 (100)	
Not assessable, n (%)	0 (0.0)	0 (0.0)	0 (0.0)	
Valid certificate, n (%)	4 (7.6)	7 (10.6)	7 (11.9)	0.741
**JAMA score**				
Assessable, n (%)	53 (100)	66 (100)	59 (100)	
Not assessable, n (%)	0 (0.0)	0 (0.0)	0 (0.0)	
Median (range)	3 (1-4)	3 (0-4)	3 (0-4)	0.846
**Flesch Reading Ease score**				
Assessable, n (%)	50 (94.3)	58 (87.9)	52 (88.1)	
Not assessable, n (%)	3 (5.7)	8 (12.1)	7 (11.9)	
Mean (SD)	48 (9)	48 (10)	47 (9)	0.889
**Flesch Kincaid Grade level**				
Assessable, n (%)	44 (83.0)	53 (80.3)	50 (84.8)	
Not assessable, n (%)	9 (17.0)	13 (19.7)	9 (15.3)	
Mean (SD)	12 (2)	11 (3)	12 (2)	0.853
**Sum DISCERN score**				
Assessable, n (%)	52 (98.1)	62 (93.9)	56 (94.9)	
Not assessable, n (%)	1 (1.9)	4 (6.1)	3 (5.1)	
All websites, median (range)	29 (20-43)	28 (16-43)	29 (16-43)	0.204
Top 10 website hits, median (range)	31 (23-43)	32 (20-38)	33 (23-38)	0.837
**Sum key fact score**				
Assessable, n (%)	53 (100)	66 (100)	59 (100)	
Not assessable, n (%)	0 (0.0)	0 (0.0)	0 (0.0)	
All websites, median (range)	16 (4-37)	14 (3-37)	13 (3-37)	0.773
Top 10 website hits, median (range)	13 (9-31)	14 (6-37)	19 (6-37)	0.771
**Misleading/wrong facts**				
Websites with misleading/wrong facts, n (%)	3 (5.7)	8 (12.1)	8 (13.6)	0.357
Overall identified wrong facts, n	11	35	35	

[a] Scientific/governmental versus foundation/advocacy organizations versus other.
[b] Europe versus North America.
HON, Health on the Net; JAMA, Journal of the American Medical Association; SD, standard deviation.
