# Supplementary material for: Accuracy and Reliability of Internet Resources for Information on Monoclonal Gammopathy of Undetermined Significance—What Information Is out There for Our Patients?
Source: Cancers (Basel). 2021 Sep 7;13(18):4508. doi: 10.3390/cancers13184508 (PMC8465467; doi:10.3390/cancers13184508)
Supplement: Supplementary file 1 [file cancers-13-04508-s001.zip › cancers-1361372-supplementary/Supplementary Material/Table S4.pdf]

Table S4: DISCERN score for websites and videos.

Health information source	A. Websites		B. Videos
**Overall unique websites/videos, n (%)**	86 (100)		61 (100)
**Assessable, n (%)**	84 (97.8)		61 (100)
**Not assessable, n (%)**	2 (2.3)		0 (0.0)
**Section 1: Is the publication reliable? (item 1-8), median (range)**			
Explicit aims	2 (1-4)		2 (1-4)
Aims achieved	2 (2-5)		3 (1-4)
Relevance to patients	3 (2-5)		3 (2-4)
Sources of information	2 (1-5)		1 (1-4)
Currency (date) of information	2 (1-5)		1 (1-4)
Bias and balance	2 (1-5)		1 (1-4)
Additional sources of information	1 (1-4)		1 (1-2)
Reference to areas of uncertainty	1 (1-3)		1 (1-3)
Overall section 1	*14 (8-27)*		*14 (8-22)*
**Section 2: How good is the quality of information on treatment/follow-up choices? (item 9-15), median (range)**			
Significance of MGUS diagnosis	2 (1-4)		2 (1-4)
Benefits of follow-up	1 (1-4)		1 (1-3)
Disadvantages of follow-up	1 (1-1)		1 (1-2)
Risks of no follow-up	1 (1-2)		1 (1-2)
Uncertainty associated with follow-ups	1 (1-5)		1 (1-4)
Variation of follow-up intervalls	1 (1-2)		1 (1-3)
Shared decision making	1 (1-5)		1 (1-2)
Overall section 2	*9 (7-16)*		*9 (7-14)*
**Section 3: Overall rating of the publication (item 16), median (range)**			
Overall quality	3 (1-4)		2 (1-3)

The categorial item scoring of the DISCERN score ranges between 1 (not addressed/fulfilled) and 5 (fully addressed/fulfilled).
MGUS, monoclonal gammopathy of undetermined significance.
