# Supplementary material for: Accuracy and Reliability of Internet Resources for Information on Monoclonal Gammopathy of Undetermined Significance—What Information Is out There for Our Patients?
Source: Cancers (Basel). 2021 Sep 7;13(18):4508. doi: 10.3390/cancers13184508 (PMC8465467; doi:10.3390/cancers13184508)
Supplement: Supplementary file 1 [file cancers-13-04508-s001.zip › Supplementary Material/Table S2.docx]

**Table S2: Website and video content evaluation by MGUS key facts.**

| **Category** | **Item** | **Description** |
| --- | --- | --- |
| **Definition** | IgM MGUS | Serum IgM M protein < 30 g/L, BM lymphoplasmacytic infiltration < 10 %, no symptoms or end-organ damage that can be attributed to the lymphoproliferative disorder. |
|  | Non-IgM MGUS | Serum M protein (non-IgM type) < 30 g/L, clonal BM PCs < 10 %, absence of end-organ damage. |
|  | Light-chain MGUS | Abnormal FLC ratio, increased level of the involved LC, no heavy chain expression on immunofixation, absence of end-organ damage, clonal BM PCs <10 %, urinary M protein < 500 mg/24 h. |
|  | BM PCs < 10 % | self-explanatory |
|  | M protein < 30 g/L | self-explanatory |
|  | No end organ damage | No evidence of CRAB criteria, i.e. hyper**c**alcemia, **r**enal insufficiency, **a**nemia, **b**one lesions. |
|  | No SLiM criteria | SLiM criteria: clonal BM PCs < **s**ixty percent, involved:uninvolved serum FLC ratio < 100, ≤ 1 focal lesions on MRI studies. |
|  | Diagnosis of exclusion | No diagnostic criteria of other hemato-oncological entities met. |
| **Symptoms** | None | No evidence of symptoms that can be attributed to MGUS. |
|  | Incidental finding | MGUS is often diagnosed incidentally. |
| **Risk factors** | Age | Incidence increases with age. |
|  | Male | Slightly higher incidence in men compared to women. |
|  | First degree relative | Increased risk in case of first-degree relatives diagnosed with MGUS. |
| **Evaluation** | Medical history | Mandatory to obtain any relevant information on patient's health status. |
|  | Clinical examination | Mandatory to assess any signs or symptoms of a medical condition. |
|  | Differential blood count | Differentiation of white blood cells. |
|  | Electrolytes | Sodium, potassium, calcium etc. |
|  | Kidney retention parameters | Creatinine, glomerular filtration rate, urea. |
|  | Total protein, albumin (S) | Quantity of total protein and albumin in serum. |
|  | Protein electrophoresis (S) | Separates the serum protein components into five major fractions. Examines which globulin fraction is elevated. |
|  | Immunofixation (S) | Assessment of the M protein type in serum. |
|  | Immunofixation (U) | Assessment of the M protein type in urine. |
|  | Ig (S) | Concentration of IgG, IgA, IgM in serum. |
|  | FLCs (S) | Concentration of FLCs (kappa and lambda) in serum. |
|  | 24 h urine for protein quantification | Quantifies the urinary protein excretion per 24 h. |
|  | LDH | Non-specific marker of cell turnover. |
|  | NT-proBNP | Normal level helps to rule out chronic heart failure (particularly relevant in case of AL amyloidosis). |
|  | ALAT | Evaluation of liver function. |
|  | Beta-2-microglobulin (S) | (Prognostic) marker in PC disorders, particularly MM. |
|  | Low dose whole body CT | Detection of osteolyses and osteopenia, without contrast agents. |
|  | Radiography not a standard | Projection radiography is no longer standard of diagnostics in PC disorders. |
|  | MRI | Evaluation of diffuse BM infiltration, focal bone lesions and extramedullary manifestations. |
|  | BM cytology | Assessment of extent of PC infiltration and status of other hematopoietic cells. |
|  | BM histology | Assessment of extent of PC infiltration and status of other hematopoietic cells in the context of BM stroma. |
|  | BM cytogenetics/FISH | Detection of chromosomal aberrations. |
| **Management** | No treatment indication | No treatment indication is given when MGUS diagnosis is established and differential diagnoses or organ damage are excluded. |
|  | Continuous follow-up | Continuous follow-up is mandatory to detect disease progression or organ damage and initiate treatment. |
|  | Risk factors guided follow-up | Follow-up intervals might be defined in dependency of presence/absence of risk factors. |
| **Outcome** | Precancerous condition | MGUS can progress into a hemato-oncological disorder and end organ damage might develop, i.e. smoldering MM, MM, B-NHL, MGRS, neuropathy, AL amyloidosis. |
|  | Smoldering MM | Serum M protein ≥ 30 g/L or urinary M protein ≥ 500 mg/24 h or BM PCs ≥ 10-60 %, absence of MM defining events or AL amyloidosis. |
|  | MM | Clonal BM PCs ≥ 10% or biopsy proven plasmacytoma and at least one of the CRAB or SLiM criteria. |
|  | B-NHL | Particularly in case of IgM-MGUS is B-NHL the underlying disease that might require treatment in case of progression. |
|  | MGRS | Monoclonal gammopathy with unclear renal insufficiency or significant proteinuria. Criteria of multiple myeloma or other lymphoproliferative diseases not met. |
|  | Neuropathy | Deposits of non-functional M protein at peripheral nerves can cause neuropathy. |
|  | AL amyloidosis | Deposits of non-functional M protein can affect nearly every organ and cause severe organ damage. |
| **Risk of progression** | LR MGUS | Serum M protein < 15 g/L, normal FLC ratio. |
|  | IR/HR MGUS | All other than LR MGUS. |
|  | Progression rate LR MGUS | Approximately 5 % in 20 years. |
|  | Progression rate IR/HR MGUS | 20-60 % in 20 years. |
|  | Per year MM progression rate | The risk to develop a MM or another lymphoproliferative disorder is approximately 1 % per year. |

MGUS key facts were sourced from Blood guideline “How I manage monoclonal gammopathy of undetermined significance”, International Myeloma Working Group updated criteria for the diagnosis of multiple myeloma [and MGUS]. and Deutsche Gesellschaft für Hämatologie und Medizinische Onkologie e. V. (DGHO) on MGUS.^1-3^

AL-amyloidosis, light chain-amyloidosis; ALAT, alanine aminotransferase; BM, bone marrow; B-NHL, B-cell non-Hodgkin lymphoma; CT, computer tomography; FLC, free light chain; FISH, fluorescence in-situ hybridization; HR, high-risk; Ig, immunoglobulin; IR, intermediate-risk; LC, light chain; LDH, lactate dehydrogenase; LR, low-risk; MGRS, monoclonal gammopathy of renal significance; MGUS, monoclonal gammopathy of undetermined significance; MM, multiple myeloma; M protein, monoclonal protein; MRI, magnetic resonance imaging; NT-proBNP,  N-terminal pro b-type natriuretic peptide; PC, plasma cell; S, serum; U, urine.

1. Go RS, Rajkumar SV. How I manage monoclonal gammopathy of undetermined significance. *Blood* 2018; **131**(2)**:** 163-173. e-pub ahead of print 2017/12/01; doi: 10.1182/blood-2017-09-807560

2. Rajkumar SV, Dimopoulos MA, Palumbo A, Blade J, Merlini G, Mateos MV *et al.* International Myeloma Working Group updated criteria for the diagnosis of multiple myeloma. *Lancet Oncol* 2014; **15**(12)**:** e538-548. doi: 10.1016/S1470-2045(14)70442-5

3. Scheid C, Driessen C, Knop S, Krauth MT, Naumann R, Schieferdecker A *et al.* Monoklonale Gammopathie unklarer Signifikanz (MGUS). In: DGHO, 2019.
