# Supplementary material for: Accuracy and Reliability of Internet Resources for Information on Monoclonal Gammopathy of Undetermined Significance—What Information Is out There for Our Patients?
Source: Cancers (Basel). 2021 Sep 7;13(18):4508. doi: 10.3390/cancers13184508 (PMC8465467; doi:10.3390/cancers13184508)
Supplement: Supplementary file 1 [file cancers-13-04508-s001.zip › Supplementary Material/Table S3.docx]

**Table S3: Characterization of websites by search engine.**

| **Search engine** | **Google** | **Bing** | **Yahoo** | ***P* value** |
| --- | --- | --- | --- | --- |
| **Websites, n** | 53 | 66 | 59 |  |
| **Website category, n (%)** |  |  |  |  |
| Scientific/governmental | 24 (45.3) | 30 (45.5) | 29 (49.2) | 0.096^a^ |
| Foundation/advocacy | 15 (28.3) | 27 (40.9) | 25 (42.4) |  |
| News/media | 12 (22.6) | 5 (7.6) | 3 (5.1) |  |
| Industry/for profit | 2 (3.8) | 2 (3.0) | 2 (3.4) |  |
| Personal commentary/blog | 0 (0.0) | 2 (3.0) | 0 (0.0) |  |
| **Host continent, n (%)** |  |  |  |  |
| Europe | 7 (13.2) | 7 (10.6) | 7 (11.9) | 0.937^b^ |
| North America | 43 (81.1) | 53 (80.3) | 47 (79.7) |  |
| South America | 1 (1.9) | 0 (0.0) | 0 (0.0) |  |
| Asia | 0 (0.0) | 1 (1.5) | 0 (0.0) |  |
| Australia | 2 (3.8) | 3 (4.5) | 3 (5.1) |  |
| Africa | 0 (0.0) | 0 (0.0) | 0 (0.0) |  |
| Antarctica | 0 (0.0) | 0 (0.0) | 0 (0.0) |  |
| Not assessable | 0 (0.0) | 2 (3.0) | 2 (3.4) |  |
| **HON foundation certificate** |  |  |  |  |
| Assessable, n (%) | 53 (100) | 66 (100) | 59 (100) |  |
| Not assessable, n (%) | 0 (0.0) | 0 (0.0) | 0 (0.0) |  |
| Valid certificate, n (%) | 4 (7.6) | 7 (10.6) | 7 (11.9) | 0.741 |
| **JAMA score** |  |  |  |  |
| Assessable, n (%) | 53 (100) | 66 (100) | 59 (100) |  |
| Not assessable, n (%) | 0 (0.0) | 0 (0.0) | 0 (0.0) |  |
| Median (range) | 3 (1-4) | 3 (0-4) | 3 (0-4) | 0.846 |
| **Flesch Reading Ease score** |  |  |  |  |
| Assessable, n (%) | 50 (94.3) | 58 (87.9) | 52 (88.1) |  |
| Not assessable, n (%) | 3 (5.7) | 8 (12.1) | 7 (11.9) |  |
| Mean (SD) | 48 (9) | 48 (10) | 47 (9) | 0.889 |
| **Flesch Kincaid Grade level** |  |  |  |  |
| Assessable, n (%) | 44 (83.0) | 53 (80.3) | 50 (84.8) |  |
| Not assessable, n (%) | 9 (17.0) | 13 (19.7) | 9 (15.3) |  |
| Mean (SD) | 12 (2) | 11 (3) | 12 (2) | 0.853 |
| **Sum DISCERN score** |  |  |  |  |
| Assessable, n (%) | 52 (98.1) | 62 (93.9) | 56 (94.9) |  |
| Not assessable, n (%) | 1 (1.9) | 4 (6.1) | 3 (5.1) |  |
| All websites, median (range) | 29 (20-43) | 28 (16-43) | 29 (16-43) | 0.204 |
| Top 10 website hits, median (range) | 31 (23-43) | 32 (20-38) | 33 (23-38) | 0.837 |
| **Sum key fact score** |  |  |  |  |
| Assessable, n (%) | 53 (100) | 66 (100) | 59 (100) |  |
| Not assessable, n (%) | 0 (0.0) | 0 (0.0) | 0 (0.0) |  |
| All websites, median (range) | 16 (4-37) | 14 (3-37) | 13 (3-37) | 0.773 |
| Top 10 website hits, median (range) | 13 (9-31) | 14 (6-37) | 19 (6-37) | 0.771 |
| **Misleading/wrong facts** |  |  |  |  |
| Websites with misleading/wrong facts, n (%) | 3 (5.7) | 8 (12.1) | 8 (13.6) | 0.357 |
| Overall identified wrong facts, n | 11 | 35 | 35 |  |

^a^ Scientific/governmental versus foundation/advocacy organizations versus other.

^b^ Europe versus North America.

HON, Health on the Net; JAMA, Journal of the American Medical Association; SD, standard deviation.
