# Supplementary material for: Accuracy and Reliability of Internet Resources for Information on Monoclonal Gammopathy of Undetermined Significance—What Information Is out There for Our Patients?
Source: Cancers (Basel). 2021 Sep 7;13(18):4508. doi: 10.3390/cancers13184508 (PMC8465467; doi:10.3390/cancers13184508)
Supplement: Supplementary file 1 [file cancers-13-04508-s001.zip › Supplementary Material/Table S4.docx]

**Table S4: DISCERN score for websites and videos.**

| **Health information source** | **A. Websites** |  | **B. Videos** |
| --- | --- | --- | --- |
| **Overall unique websites/videos, n (%)** | 86 (100) |  | 61 (100) |
| **Assessable, n (%)** | 84 (97.8) |  | 61 (100) |
| **Not assessable, n (%)** | 2 (2.3) |  | 0 (0.0) |
| **Section 1: Is the publication reliable? (item 1-8), median (range)** | | | |
| Explicit aims | 2 (1-4) |  | 2 (1-4) |
| Aims achieved | 2 (2-5) |  | 3 (1-4) |
| Relevance to patients | 3 (2-5) |  | 3 (2-4) |
| Sources of information | 2 (1-5) |  | 1 (1-4) |
| Currency (date) of information | 2 (1-5) |  | 1 (1-4) |
| Bias and balance | 2 (1-5) |  | 1 (1-4) |
| Additional sources of information | 1 (1-4) |  | 1 (1-2) |
| Reference to areas of uncertainty | 1 (1-3) |  | 1 (1-3) |
| *Overall section 1* | *14 (8-27)* |  | *14 (8-22)* |
| **Section 2: How good is the quality of information on treatment/follow-up choices? (item 9-15), median (range)** | | | |
| Significance of MGUS diagnosis | 2 (1-4) |  | 2 (1-4) |
| Benefits of follow-up | 1 (1-4) |  | 1 (1-3) |
| Disadvantages of follow-up | 1 (1-1) |  | 1 (1-2) |
| Risks of no follow-up | 1 (1-2) |  | 1 (1-2) |
| Uncertainty associated with follow-ups | 1 (1-5) |  | 1 (1-4) |
| Variation of follow-up intervalls | 1 (1-2) |  | 1 (1-3) |
| Shared decision making | 1 (1-5) |  | 1 (1-2) |
| *Overall section 2* | *9 (7-16)* |  | *9 (7-14)* |
| **Section 3: Overall rating of the publication (item 16), median (range)** | | | |
| Overall quality | 3 (1-4) |  | 2 (1-3) |

The categorial item scoring of the DISCERN score ranges between 1 (not addressed/fulfilled) and 5 (fully addressed/fulfilled).

MGUS, monoclonal gammopathy of undetermined significance.
